# Effect of Codonopsis Radix and Polygonati Rhizoma on the regulation of the IRS1/PI3K/AKT signaling pathway in type 2 diabetic mice

**DOI:** 10.3389/fendo.2022.1068555

**Published:** 2022-12-14

**Authors:** Yong-po Mao, Yi-ming Song, Sheng-wang Pan, Ning Li, Wen-xiang Wang, Bin-bin Feng, Jian-hai Zhang

**Affiliations:** ^1^ School of Pharmacy, Chongqing Three Gorges Medical College, Chongqing, China; ^2^ School of Food and Biological Engineering, Chengdu University, Chengdu, China; ^3^ Chongqing Key Laboratory of Development and Utilization of Genuine Medicinal Materials in Three Gorges Reservoir Area, Chongqing Three Gorges Medical College, Chongqing, China; ^4^ Chongqing Engineering Research Center of Antitumor Natural Drugs, Chongqing Three Gorges Medical College, Chongqing, China

**Keywords:** type 2 diabetes mellitus, pathology section, western blotting, hypoglycemic, Codonopsis Radix and Polygonati Rhizoma

## Abstract

**Objective:**

Codonopsis Radix and Polygonati Rhizoma (CRPR) has a good hypoglycemic effect. The aims of the present study were to investigate the effect of CRPR on high-fat/high-sugar diet (HFHSD)- and streptozotocin (STZ)-induced type 2 diabetes mellitus (T2DM) mice as well as to investigate the involved mechanism.

**Methods:**

A T2DM mouse model was generated by combining HFHSD and STZ. After the model was established, normal and model groups received the same volume of normal saline intragastrically, and the negative control group was treated with metformin (200 mg/kg·BW). The low, medium, and high CRPR groups received four consecutive weeks of oral gavage with CRPR doses of 2.5, 5, and 10 g/kg·BW, respectively, during the course of the study. Body weight and fasting blood glucose (FBG) were measured on a weekly basis. Enzyme-linked immunosorbent assay (ELISAs) were used to evaluate the serum and liver samples. Hematoxylin and eosin (H&E) staining was utilized to observe the pathological status of the liver and pancreas. Western blot (WB) analysis was performed to evaluate the protein expression levels of PI3K, p-PI3K, AKT, and p-AKT.

**Results:**

Compared to model mice, each treatment group had significantly elevated levels of FBG, total cholesterol (TC), and triacylglycerol (TG) (*P<*0.01 and *P<*0.05, respectively). The levels of alanine aminotransferase (ALT) and aspartate aminotransferase (AST) were significantly reduced in the treatment groups compared to the model group (*P<*0.01). Compared to the model group, fasting insulin (FINS) levels were elevated in all groups of CRPR (*P<*0.05), and there were significantly higher levels of high-density lipoprotein cholesterol (HDL-C) in both the low-dose and high-dose CRPR groups (*P<*0.05). H&E staining indicated that CRPR treatment reduced organ enlargement, improved liver lipid accumulation, and repaired islet injury in T2DM mice. Moreover, WB analysis demonstrated that all CRPR groups significantly upregulated the protein expression of IRS1, p-GSK3β, PI3K, p-Akt and p-FOXO1(*P<*0.05) as well as significantly downregulated p-IRS1 and FOXO1 protein expression (*P*<0.05).

**Conclusion:**

The present study demonstrated that CRPR effectively improves the metabolic disturbance of lipids, repairs damaged liver tissues, repairs damaged pancreatic tissues, and reduces insulin resistance (IR) in T2DM mice. The mechanism of action may be associated with upregulation of the IRS1/PI3K/AKT signaling pathway and inhibition of IRS1 phosphorylation.

## 1 Introduction

Type 2 diabetes mellitus (T2DM) is a metabolic disease with a high disease rate, accounting for 90% of diabetes mellitus (DM) patients. As one of the world’s most serious health risks, it has many complications (1). T2DM is a chronic disease with multiple etiologies leading to insulin resistance (IR), the main pathophysiological mechanism of T2DM that is present throughout the course of T2DM. Insulin regulates carbohydrate, lipid, and protein metabolism through typical insulin signaling, including insulin receptors, insulin receptor substrate proteins, PI3K, and Akt; IR results in insufficient insulin secretion or the body’s inability to effectively use insulin, which leads to continuous increase of blood glucose levels (2).

Chinese medicine has a long history of treating DM, which is considered to belong to the category of “*Xiao Ke*”, a condition of “dryness-heat due to deficiency of yin” (3–5). Codonopsis Radix (CR) has the functions of invigorating and supplementing *Qi* and spleen as well as tonifying lung, nourishing blood, and engendering liquid. Researchers have found that *Codonopsis pilosula* polysaccharide lowers blood glucose levels and improves insulin sensitivity in diabetic rats (6, 7). In Chinese medicine, Polygonati Rhizoma (PR) has the therapeutic functions of reinforcing *Qi* and nourishing yin as well as invigorating the function of the spleen, moistening the lung, and benefitting the kidney. Studies have shown that PR has good hypoglycemic properties (8). *Polygonatum sibiricum* polysaccharide and saponin are effective bioactive compounds in the treatment of T2DM, showing significant anti-hyperglycemic activity in streptozotocin (STZ)-induced mice, utlimately improving insulin tolerance and affecting lipid metabolism (9, 10). In a previous study, network pharmacology found that Codonopsis Radix-Polygonati Rhizoma (CRPR) jointly affects the PI3K/Akt signaling pathway as a bridge to alleviate or improve IR in T2DM (11). In the present study, we investigated the mechanism of CRPR improving T2DM, which may be closely related to the IRS1/PI3K/Akt signaling pathway.

## 2 Materials and methods

### 2.1 Materials

The following materials were used in the present study: CR and PR (2010001 and 1912001, respectively, Shennv Pharmaceutical, Chongqing, China); high-fat/high-sugar diet (HFHSD) (Boaigang, Beijing, China; 66.5% basic feed, 10% lard, 20% sucrose, 1% cholesterol, 1% bile salt, 1% mineral mixture, and 0.5% vitamin mixture); STZ (Boaigang, purity: 98%, CAS:18883-66-4); mouse total cholesterol (TC) assay kit (A111-1-1, Jiancheng, Nanjing); mouse triglyceride (TG) assay kit (A110-1-1, Jiancheng, Nanjing); mouse high-density lipoprotein cholesterol (HDL-C) assay kit (A112-1-1, Jiancheng, Nanjing); mouse low-density lipoprotein cholesterol (LDL-C) assay kit (A113-1-1, Jiancheng, Nanjing); mouse alanine aminotransferase (ALT) assay kit (C009-2-1, Jiancheng, Nanjing); mouse aspartate aminotransferase (AST) assay kit (C010-2-1, Jiancheng, Nanjing); mouse insulin enzyme-linked reaction kit (H203-1-2, Jiancheng, Nanjing); phospho-IRS1 (S323) polyclonal antibody (Source: rabbit, Cat. No. BS4271, Bioworld Technology); IRS1(D606) polyclonal antibody (Source: Rabbit, Cat. No. BS9113, Bioworld Technology); phospho-GSK3α/β (Y279/216) polyclonal antibody (Source: rabbit, Cat. No. BS4083, Bioworld Technology); GSK3α/β (G273) polyclonal antibody (Source: rabbit, Cat. No. BS1412, Bioworld Technology); phospho-PI3K p85α (Tyr607) polyclonal antibody (Source: rabbit, Cat. No. AP0153, Bioworld Technology); PI3K p85α (Q498) polyclonal antibody (Source: rabbit, Cat. No. BS3678, Bioworld Technology); phospho-Akt (S473) polyclonal antibody (Source: rabbit, Cat. No. BS4006, Bioworld Technology); Akt polyclonal antibody (Source: rabbit, Cat. No. AP0059, Bioworld Technology); *β*-actin (MG3) monoclonal antibody (Source: mouse, Cat. No. A0101, Lablead); BCA protein assay kit (B5000, Lablead); Marker (10-180Kd, P1018, Lablead); goat anti-mouse IgG(H+L)-HRP (S0100, Lablead); ECL (E1070, Lablead); radioimmunoprecipitation assay (RIPA; Strong; R1091, Lablead); TBST (T9039, Lablead); SDS-PAGE sample loading buffer (P0015L, Beyotime, 5 ×); and polyvinylidene difluoride (PVDF) membranes (Millipore, IPVH00010).

### 2.2 Experimental animals

Eight-week-old male Kunming mice in good health, weighing 35 ± 3 g, were purchased from Hunan Slake Jing da Experimental Animal Co., Ltd. (Hunan, China; license number SCXK (Xiang) 2019-0004). All mice were group housed with 5 animals per cage in a sanitary environment (temperature of 24 ± 2 °C, 50 ± 10% humidity, and 12-h light/dark cycle), and they were provided standard pellet diet and drinking water.

### 2.3 Establishment of T2DM mouse models

In total, 60 mice were randomly divided into a normal control group (NC) and model groups. The NC group was given normal chow, and the model groups were given HFHSD chow. After 8 weeks on a HFHSD to induce IR, the model group received intraperitoneal injections of STZ (75 mg/kg per injection for 2 d). The NC group received the same volume of sodium citrate buffer solution. At Day 7 after the final injection, blood was collected from the mice to test the fasting blood glucose (FBG) values. T2DM model mice with FBG levels reaching or exceeding 11.1 mmol/L (12) in two consecutive tests indicated successful modelling.

### 2.4 Preparation of CRPR extract

Based on previously reported methods (13) and the pre-test results, the CRPR formula consisted of a 1.5:2 ratio. Seven volumes of purified water were added to the first extraction and heated, and once the water boiled, the mixture was decocted for 45 min. The residue was filtered, and the filtrate was subjected to a second extraction, in which 3 volumes of purified water was added followed by the same process as in the previous step. Utilizing a rotary evaporator, the filtrate was concentrated to a relative density of 1.1-1.3 and set aside.

### 2.5 Animal grouping and drug administration

A random sample of successful model mice was divided into five groups as follows: model (MC) group, metformin (MET) group, CRPR low-dose (CRPR-L) group, CRPR medium-dose (CRPR-M) group, and CRPR high-dose (CRPR-H) group. The low-, medium-, and high-dose groups were given 2.5, 5, and 10 g/kg·BW CRPR by oral gavage (1.05 g/kg for adults per dose), respectively, and the MET group was given 200 mg/kg of metformin solution (14). The MC and NC groups received the same volume of saline for four weeks.

### 2.6 Determination of hypoglycemic activity of CRPR

Every 7 days during the administration period, body weight and FBG values were measured after the mice were fasted for 12 h. At the end of the experiment, mice were given a 20% glucose solution (2 g/kg) *via* gavage after fasting overnight, and a glucometer was then used to measure blood glucose levels at 0, 30, 60, and 120 min from the tail vein and recorded as a, b, c, and d, respectively. The area under the curve (AUC) (15) was then calculated using the following equation:


$$AUC=\frac{\left(a+b\right)*0.5}{2}+\frac{\left(b+c\right)*0.5}{2}+\frac{\left(c+d\right)*0.5}{2}$$

With an insulin ELISA kit, fasting serum insulin (FINS) was determined in mice. The homeostasis model assessment-insulin resistance (HOMA-IR) index was then calculated from FBG and FINS (16) using the following formula:


$$HOMA-IR=\frac{FINS\left(mIU/L\right)*FBG\left(mmol/L\right)}{22.5}$$

### 2.7 Determination of biochemical parameters

Following the final injection, mice were fasted for 12 h, and femoral artery blood was collected. The blood was left for 15-30 min at room temperature and centrifuged at 4000 r/min for 30 min at 4°C, and the serum was separated and then stored at -80 °C. The TC, TG, LDL-C, HDL-C, AST, and ALT levels were measured and calculated according the instructions of the kits.

### 2.8 Determination of organ indices and histopathological observation

After blood collection, liver and pancreatic tissues were collected from each group of mice, washed with saline, and weighed, and the organ indices were calculated (17). The tissues and organs were fixed with 4% paraformaldehyde, dehydrated in gradient ethanol, embedded in paraffin, cut into 5-μm serial sections, and stained with hematoxylin-eosin stain (H&E) (18). Observations were conducted under an inverted microscope on some tissue sections, and the other tissue sections were stored at -80°C.

### 2.9 Western blot analysis

Liver tissue (100 mg) was homogenized in RIPA lysis buffer containing phenylmethylsulfonyl fluoride (PMSF) and incubated in an ice bath for 30 min. The sample was then centrifuged at 12000 r/min for 30 min at 4°C, and the supernatant was collected to assess the total protein concentration using a bicinchoninic acid (BCA) assay kit. Protein loading buffer was added to the sample for a final concentration of 3 μg/μL, and the sample was denatured by heating and stored at -20°C for later use. An SDS-PAGE kit was used to prepare a 10% separating gel and 5% stacking gel. Marker (5 μL) and protein samples (10 μL) were loaded into the gel, electrophoresed, and then transferred to PVDF membranes. After washing and blocking for 15 min, the PVDF membranes were incubated overnight at 4 °C with the following primary antibodies: anti-p-IRS1 (1:1000), anti-IRS1 (1:1000), anti-p-GSK3β (1:1000), anti-GSK3β (1:1000), anti-p-PI3K (1:1000), anti-PI3K (1:1000), anti-p-Akt (1:1000), anti-Akt(1:1000), and anti-*β*-actin (1:2000). The membranes were then washed three times with TBST followed by incubation with goat anti-rabbit secondary antibodies (1:2000) for 1.5 h. After washing with TBST, the protein bands were visualized by adding ECL reagent and imaged using a gel imager, and the grey scale values of the protein bands were analyzed by Image J software (National Institutes of Health, America, Bethesda).

### 2.10 Statistical analysis

All data are presented as the mean ± standard deviation (SD). SPSS 18.0 and GraphPad Prism 6.0 statistical software were utilized for data analyses and to generate graphs. One-way analysis of variance (ANOVA) was used for comparisons among multiple groups. Groups meeting variance homogeneity were tested by the least significant difference method (LSD), and when the variance was not homogeneous, a non-parametric test was used. *P*<0.05 and *P*<0.01 indicated significant differences.

## 3 Results

### 3.1 Effect of CRPR on general condition, body weight, and FBG

**Table 1** shows the body weight change over time. Compared to the NC group, the body weight decreased over time (*P*<0. 01) in the MC group, but the body weight gradually increased over time in the MET group. After 4 weeks of CRPR intervention, all CRPR groups showed different degrees of body weight reduction, especially the CRPR-H group (*P*<0.01), compared to the MC group.

**Table 1:** Changes of body weight in T2DM mice.

Group	Dose	Body weight (g)			
		1 week	2 weeks	3 weeks	4 weeks
NC	–	47.83 ± 3.80	51.12 ± 1.10	51.38 ± 1.51	50.66 ± 1.56
MC	–	49.55 ± 2.24	46.16 ± 1.69^**^	43.21 ± 1.15^**^	41.55 ± 1.23^**^
MET	200 mg/kg	48.95 ± 2.61	46.81 ± 2.06	45.04 ± 2.57	43.11 ± 2.69
CRPR-L	2.5 g/kg	46.60 ± 1.56^#^	43.42 ± 2.33	45.04 ± 1.88	45.42 ± 1.80^##^
CRPR-M	5 g/kg	48.62 ± 3.15	47.02 ± 3.33	48.14 ± 2.71^##^	49.64 ± 3.58^##^
CRPR-H	10 g/kg	46.12 ± 1.9^##^	43.55 ± 1.79	43.54 ± 2.63	45.91 ± 2.63^#^

All values are presented as the mean ± SD. **P<0.01 vs. the NC group; ^#^P<0.05 and ^##^P<0.01 vs. the MC group.

The effect of CRPR on FBG concentrations in mice is shown in **Table 2**. The FBG of mice in the NC group remained unchanged (5-7 mmol/L) throughout the experiment. Compared to the NC group, the FBG was significantly increased in the MC group (*P*<0.01), and the mice in the MC group remained in a hyperglycemic state throughout the experiment. After 4 weeks of CRPR intervention, the FBG was significantly decreased in the MET group and all CRPR groups (*P*<0.01) compared to the MC group with certain quantity-effect relationship as the blood glucose was lowered as the dose decreased.

**Table 2:** Effect of CRPR on blood glucose concentration in T2DM mice.

Group	Dose	Blood glucose (mmol·L^-1^)			
		1 week	2 weeks	3 weeks	4 weeks
NC	–	8.02 ± 1.41	6.22 ± 0.88	6.25 ± 0.44	6.26 ± 1.27
MC	–	21.50 ± 4.66^**^	18.15 ± 4.88^**^	19.10 ± 4.85^**^	21.44 ± 3.9^**^
MET	200 mg/kg	15.72 ± 3.38	10.91 ± 3.32^##^	6.51 ± 1.25^##^	7.97 ± 2.86^##^
CRPR-L	2.5 g/kg	13.68 ± 1.59^#^	9.56 ± 1.77^##^	7.63 ± 1.61^##^	6.41 ± 2.07^##^
CRPR-M	5 g/kg	9.64 ± 3.92^##^	8.26 ± 4.93^##^	6.86 ± 4.20^##^	5.52 ± 3.37^##^
CRPR-H	10 g/kg	11.44 ± 1.74^##^	5.88 ± 2.59^##^	7.86 ± 3.31^##^	4.92 ± 2.10^##^

All values are presented as the mean ± SD. **P<0.01 vs. the NC group; ^#^P<0.05 and ^##^P<0.01 vs. the MC group.

### 3.2 Oral glucose tolerance test and AUC

**Figure 1:**
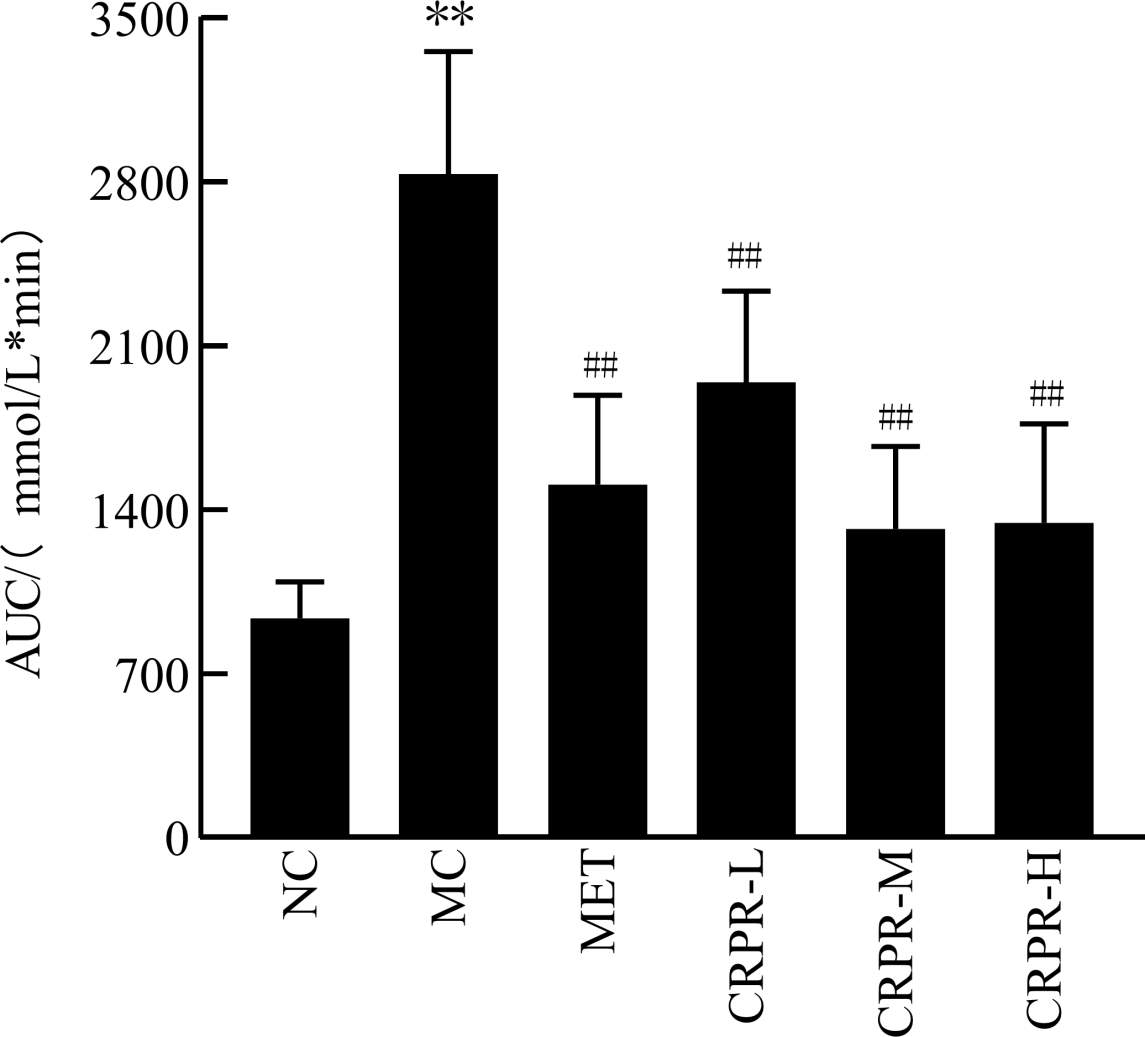
Effect of CRPR on the AUC in T2DM mice. All values are presented as the mean ± SD. **P<0.01 vs. the NC group; ^##^ *P*<0.01 vs. the MC group.

The OGTT results of CRPR on T2DM mice are shown in **Table 3**. At 120 min after detection, the blood glucose concentrations of mice in each group increased and then decreased. There was a significant difference in blood glucose levels between the MC and NC groups (*P*<0.01). The blood glucose values of each group began to decrease to near the initial value at 120 min, and the blood glucose of the mice in the CRPR-H group was significantly decreased. According to the AUC values, mice in the MC group had significantly higher blood glucose levels than mice in the NC group (*P*<0.01). In contrast, there were significant reductions in the AUC values in all CRPR groups compared to the MC group (*P*<0.01) (**Figure 1**).

**Table 3:** Effect of CRPR on glucose tolerance in T2DM mice.

Group	Dose	Blood glucose (mmol·L^-1^)			
		0 min	30 min	60 min	120 min
NC	–	6.00 ± 0.80	8.14 ± 1.69	8.88 ± 2.74	6.72 ± 1.35
MC	–	20.18 ± 5.87^**^	27.23 ± 5.40^**^	24.28 ± 4.13^**^	20.80 ± 5.94^**^
MET	200 mg/kg	7.98 ± 2.86^##^	16.72 ± 4.67^##^	13.66 ± 3.81^##^	9.01 ± 4.33^##^
CRPR-L	2.5 g/kg	7.08 ± 2.09^##^	20.06 ± 5.09^##^	17.79 ± 5.68^##^	14.56 ± 5.19^##^
CRPR-M	5 g/kg	4.26 ± 2.38^##^	15.29 ± 3.93^##^	11.97 ± 4.62^##^	8.53 ± 4.66^##^
CRPR-H	10 g/kg	4.82 ± 1.61^##^	14.88 ± 4.27^##^	12.16 ± 5.14^##^	9.31 ± 3.78^##^

All values are presented as the mean ± SD. **P<0.01 vs. the NC group; ^#^P<0.05 and ^##^P<0.01 vs. the MC group.

### 3.3 Effect of CRPR on blood lipids and liver function

**Figure 2:**
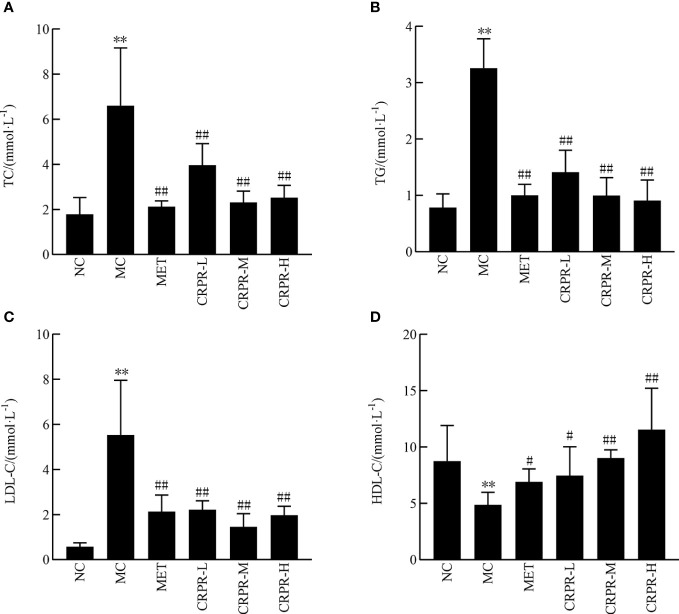
Effect of CRPR on blood lipids in T2DM mice. **(A)** Effect of CRPR on TC. **(B)** Effect of CRPR on TG. **(C)** Effect of CRPR on LDL-C. **(D)** Effect of CRPR on HDL-C. All values are presented as the mean ± SD. **P<0.01 vs. the NC group; ^#^ *P*<0.05 and ^##^ *P*<0.01 vs. the MC group.

According to the results of the mouse lipid metabolism experiment, TC, TG and LDL-C in the MC group were significantly higher than those in the NC group (*P*<0.01), but the HDL-C decreased significantly (*P*<0.01). After CRPR treatment, the serum levels of TC, TG, and LDL-C in the MET group and all CRPR groups were reduced to varying degrees compared to the MC group, among which the TG levels in the MET group and CRPR groups were significantly decreased (*P*<0.01). Compared to the MC group, the TC levels were significantly improved in the MET, CRPR-L, CRPR-M, and CRPR-H groups (*P*<0.01). Moreover, there was a statistically significant difference in HDL-C levels between the NC and MC groups (*P*<0.01), which indicated accelerated TC metabolism (**Figure 2**).

**Figure 3:**
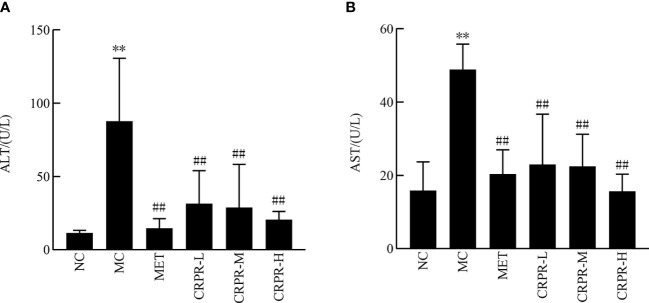
Effect of CRPR on liver function in T2DM mice. **(A)** Effect of CRPR on ALT. **(B)** Effect of CRPR on AST. All values are presented as the mean ± SD. **P<0.01 vs. the NC group; ^##^ *P*<0.01 vs. the MC group.

**Figure 3** shows the changes in liver function content among the groups. There was a significant increase in the AST and ALT contents in the MC group compared to the NC group (*P*<0.01). Moreover, the AST and ALT contents were significantly lower in the CRPR and MET groups compared to the MC group (*P*<0.01).

### 3.4 Effect of CRPR on insulin and HOMA-IR

**Figure 4:**
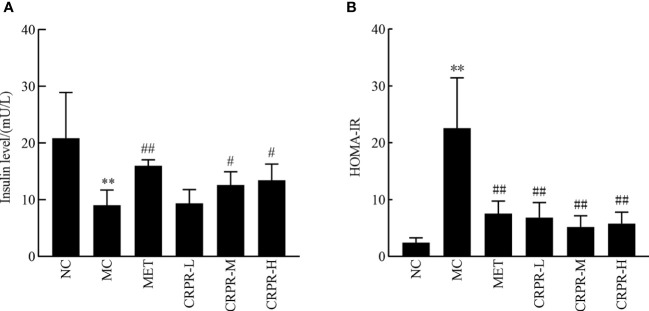
Effect of CRPR on insulin and HOMA-IR in T2DM mice. **(A)** Effect of CRPR on insulin levels. **(B)** Effect of CRPR on HOMA-IR. All values are presented as the mean ± SD. **P<0.01 vs. the NC group; ^#^ *P*<0.05 and ^##^ *P*<0.01 vs. the MC group.

Compared to the NC group, the serum insulin levels decreased in the MC group, but the IR significantly increased (**Figure 4**). Compared to the NC group, the serum insulin levels were significantly decreased in the MC group (*P*<0.01), but HOMA-IR was significantly enhanced in the MC group (*P*<0.01). Compared to the MC group, the serum insulin levels were significantly different in the CRPR-M and CRPR-H groups (*P*<0.05), and the HOMA-IR was significantly lower in the CRPR-M and CRPR-H groups.

### 3.5 Effect of CRPR on organ indices of mice

Mice in the MC group had significantly higher liver indices but significantly lower pancreatic indices compared to mice in the NC group (*P*<0.01) (**Table 4**). Compared to the MC group, the liver index was significantly decreased in the MET group and all CRPR groups (*P*<0.01). Moreover, the pancreatic index in the treatment groups decreased to different degrees compared to the MC group, but there were no statistical differences (**Table 4**).

**Table 4:** Effect of CRPR on the liver and pancreatic indices of T2DM mice.

Group	Liver index (%)	Pancreatic index (%)
NC	3.54 ± 0.47	0.93 ± 0.46
MC	6.32 ± 0.69^**^	0.47 ± 0.14^**^
MET	4.84 ± 0.28^##^	0.41 ± 0.17
CRPR-L	4.93 ± 0.61^##^	0.43 ± 0.11
CRPR-M	3.84 ± 0.53^##^	0.34 ± 0.06
CRPR-H	4.38 ± 0.21^##^	0.47 ± 0.25

All values are presented as the mean ± SD. **P<0.01 vs. the NC group; ^##^P<0.01 vs. the MC group.

### 3.6 Effect of CRPR on liver and pancreatic histopathology

**Figure 5:**
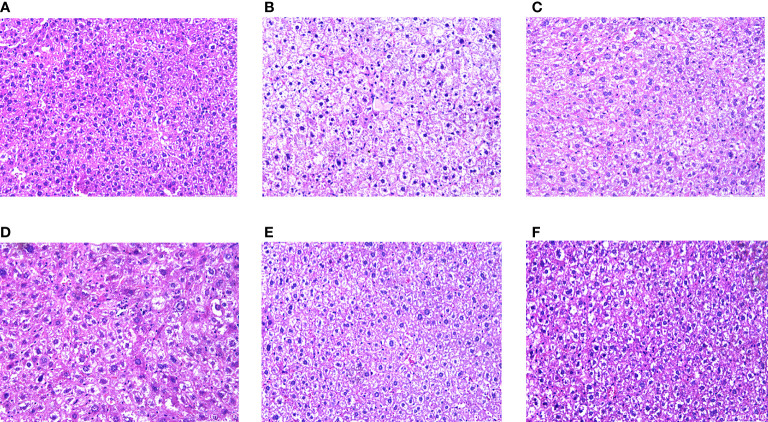
Effect of CRPR on liver histopathology. Representative H&E staining of liver sections from the **(A)** NC, **(B)** MC, **(C)** MET, **(D)** CRPR-L, **(E)** CRPR-M, and **(F)** CRPR-H groups (200x magnification).

**Figure 6:**
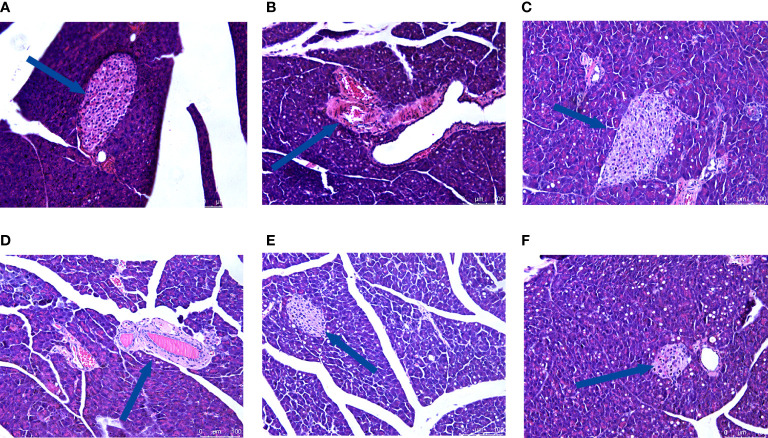
Effect of CRPR on pancreatic histopathology. Representative H&E staining of liver sections from the **(A)** NC, **(B)** MC, **(C)** MET, **(D)** CRPR-L, **(E)** CRPR-M, and **(F)** CRPR-H groups (200x magnification).

H&E staining showed that the hepatocytes in the NC group had normal morphology, normal central nucleus, and normal arrangement as well as complete and transparent tissue structure with no signs of pathology. In the MC group, however, hepatocytes showed disordered structures, and some hepatocytes showed edema, inflammatory cell infiltration, and vacuolar degeneration. Compared to the MC group, there was no obvious improvement in the CRPR-L group, but the arrangement of hepatocytes in the MET, CRPR-M, and CRPR-H groups was normal with a clear nuclear structure and reduced inflammatory cells, which indicated significant improvement in the degree of hepatic steatosis and injury. The CRPR-H group showed reduced liver tissue structural damage to a greater extent in T2DM mice compared to the other groups (**Figure 5**). Histopathology of the pancreases showed that the islets in the NC group had clear and normal structures with closely arranged islet cells and clear borders between the pancreatic alveoli and islets. In the MC group, however, the borders between the islets and alveolar cells were not obvious, and there were disordered cells, atrophied islets, and infiltrated inflammatory cells, which indicated that the pancreatic tissue was destroyed. Compared to the MC group, the borders between the exocrine glands and islets of the MET group were clearer, and the islet morphology was slightly more regular with a more uniform islet cell distribution. Moreover, the mice in the CRPR groups showed normal islet morphology with an increase in islet cells and occasional infiltration of inflammatory cells (**Figure 6**).

### 3.7 Effects of CRPR on the IRS1/PI3K/Akt pathway in liver tissues

**Figure 7:**
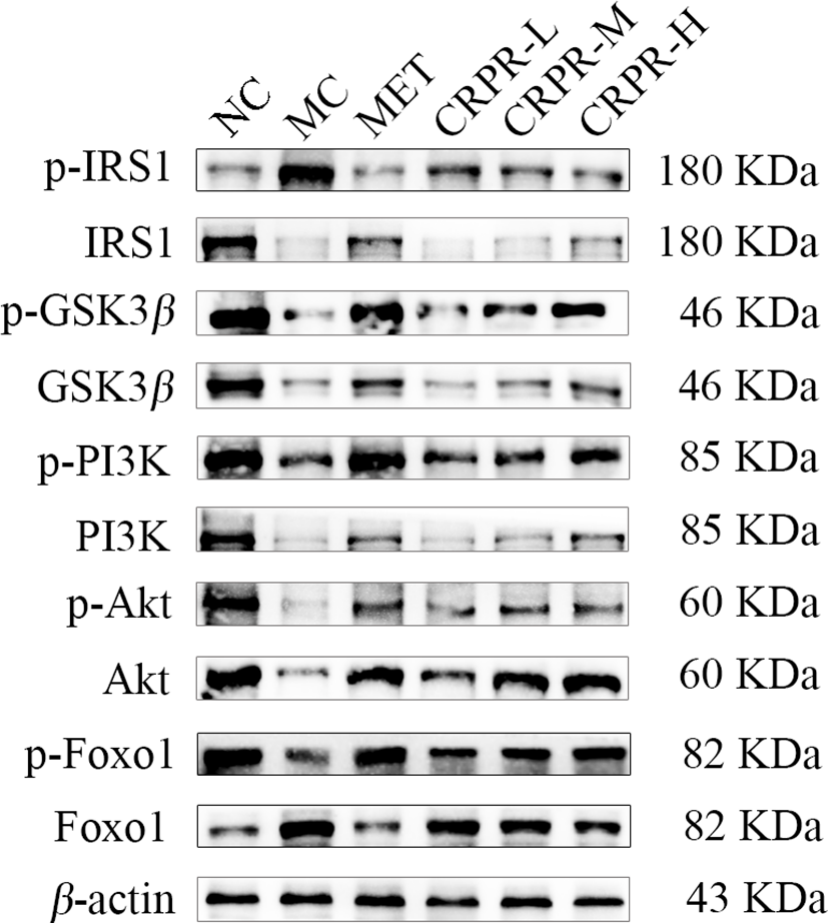
Representative Western blot analysis of p-IRS1, IRS1, p-GSK3*β*, GSK3*β*, p-PI3K, PI3K, p-Akt, Akt, p-FOXO1, and FOXO1.

**Figure 8:**
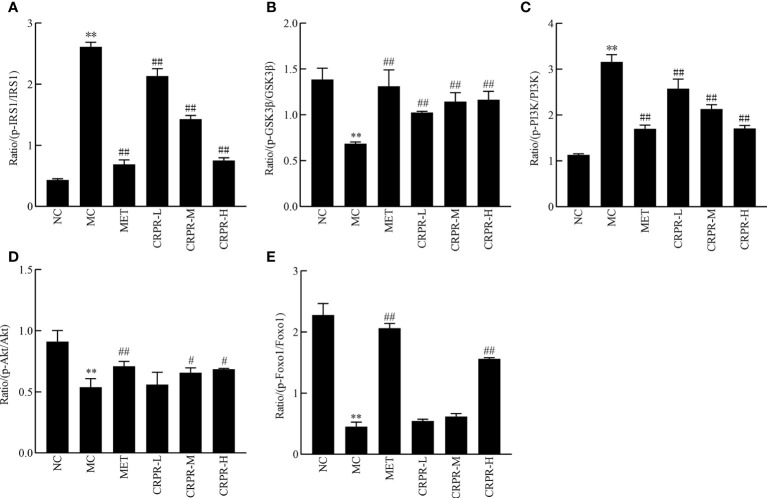
The relative expression of p-IRS1/IRS1, p-GSK3*β*/GSK3*β*, p-PI3K/PI3K, p-Akt/Akt and p-FOXO1/FOXO1. The bar graphs show the effects of CRPR on the **(A)** p-IRS1/IRS1, **(B)** p-GSK3β/GSK3β, **(C)** p-PI3K/PI3K, **(D)** p-Akt/Akt and **(E)** p-FOXO1/FOXO1 ratios. All values are presented as the mean ± SD. **P < 0.01 vs. the NC group; ^#^ *P*<0.05 and ^##^ *P* < 0.01 vs. the MC group.

To investigate effect of CRPR on the IRS1/PI3K/Akt signaling pathway in liver, the protein expression levels of p-IRS1, IRS1, p-PI3K, PI3K, p-GSK3β, GSK3β, p-Akt, and Akt in the livers were detected by WB analysis (**Figure 7**). Compared to the NC group, the p-GSK3β/GSK3β, p-Akt/Akt and p-FOXO1/FOXO1 protein expression was significantly decreased in the MC group (*P*<0.01), while the p-PI3K/PI3K and p-IRS1/IRS1 protein expression was significantly increased (*P*<0.01). Compared to the MC group, the p-GSK3β/GSK3β, p-Akt/Akt and p-FOXO1/FOXO1 ratios were significantly increased in MET and CRPR-H groups (*P*<0.05), while the p-IRS1/IRS1 and p-PI3K/PI3K protein expression was significantly decreased (*P*<0.01) in the MET, CRPR-M, and CRPR-H groups. Thus, the expressions of p-GSK3β/GSK3β and p-Akt/Akt were increased but the expressions of p-PI3K/PI3K and p-IRS1/IRS1 were decreased in each treatment group compared to the MC group (**Figure 8**).

## 4 Discussion

**Figure 9:**
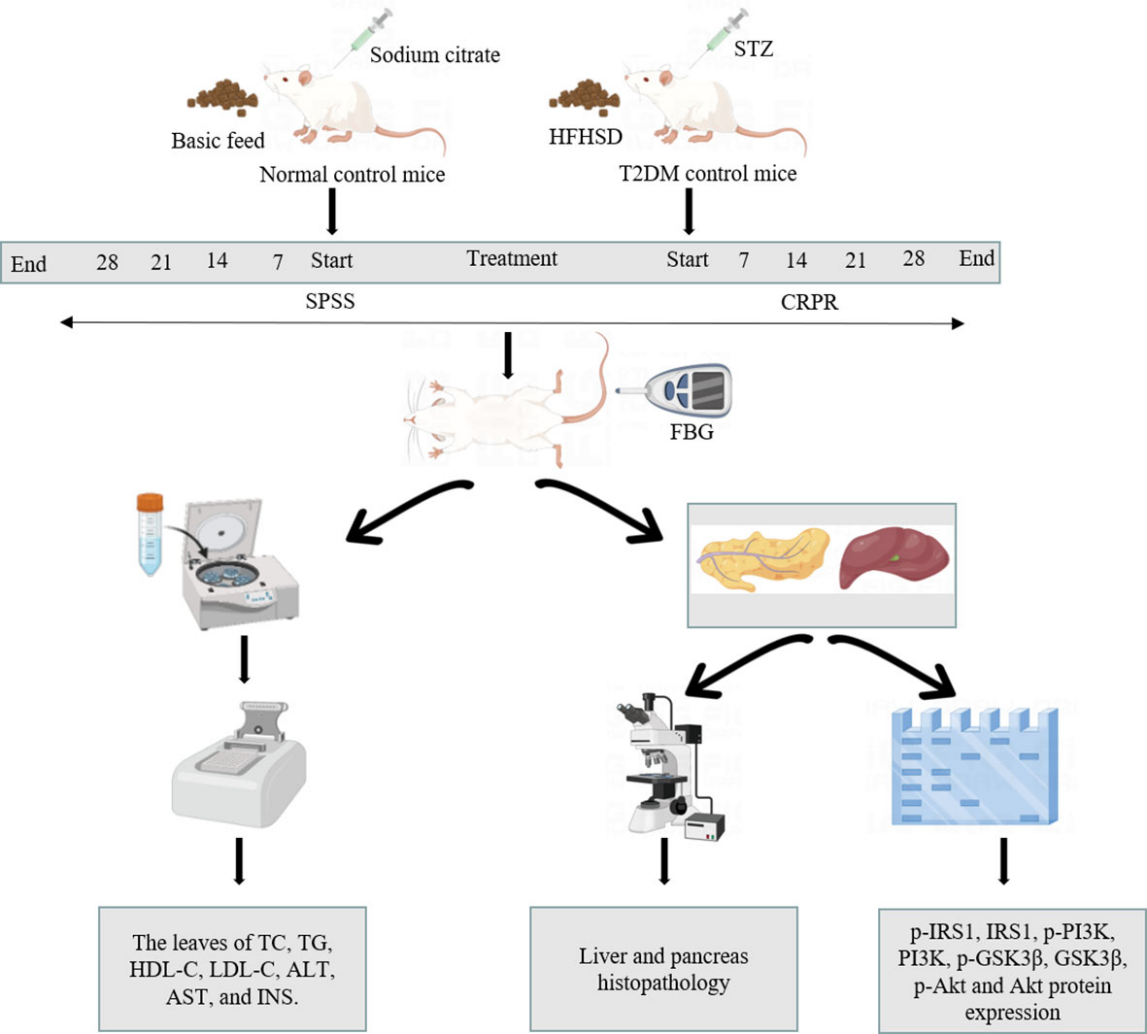
Workflow of animal study.

T2DM is caused by a dual defect of IR and insulin deficiency. Long-term IR reduces the functional defects of β cells, which in turn results in a state of reduced insulin secretion (19). In the present animal studies, mice were fed a HFHSD, which caused IR, and they were then intraperitoneally injected with STZ, which destroyed pancreatic β cells, causing T2DM. After modelling, the mice showed mental depression, polyphagia, polyuria, and significantly increased blood glucose, consistent with the symptoms reported in the literature (20), suggesting successful modelling. We then performed the animal experiments using T2DM mice as outlined in the workflow shown in **Figure 9**.

Based on a literature search and network pharmacology, we found that the active components in CRPR that improve metabolic impairment may be luteolin, baicalein, and DFV. Luteolin is a natural flavonoid with a variety of pharmacological activities (21). Luteolin inhibits the expression of TLR4 and JNK mRNA in pancreases of T2DM rats, and it improves IR and alleviates pancreatic inflammatory reactions in T2DM rats (22). Baicalein is a flavonoid found in traditional Chinese medicine, such as yellow essence and baicalensis, and it has various pharmacological effects. Cell experiments have demonstrated that baicalein reduces malondialdehyde (MDA) formation by improving antioxidant enzyme activity, which inhibits high glucose-induced oxidative stress response and PI3K/Akt signaling pathway activity, thereby reducing inflammatory factor production and blood glucose (23). DFV, a single component of dehydroalanine extracted from licorice, improves superoxide dismutase (SOD) activity and reduces MDA content, which reduces the oxidative stress damage in cardiomyocytes after high glucose stimulation (24).

The animal experiments in the present study demonstrated that the symptoms of T2DM mice in all CRPR dose groups improved after 4 weeks of continuous administration, suggesting that CRPR improves the symptoms caused by T2DM. Studies have shown that hyperglycemia causes disturbances in fat and protein metabolism in diabetic mice, leading to weight loss (25). In the present study, we showed that CRPR significantly increased body weight, significantly reduced blood glucose concentration, and significantly reduced serum insulin level in mice, indicating that CRPR improves glucose utilization in mice.

Important indicators for evaluating T2DM include FBG, FINS, OGTT, AUC, and HOMA-IR. The OGTT is a glucose loading test, which is used to investigate the body’s regulation and tolerance of glucose (26). The AUC of the OGTT curve represents the level of sugar tolerance with higher values indicating more serious damage to sugar tolerance (27). In healthy individuals, blood lipid levels are within a certain range, and blood lipid levels that exceed the normal range can lead to the development of certain diseases. With the increase of TC, TG, and LDL-C contents, the probability of suffering from diabetes, coronary heart disease, low liver function, and other diseases also increases. However, HDL-C is the opposite as it is a protective protein. When HDL-C decreases, its protective effect decreases, resulting in the occurrence of appellate diseases. Based on the results, all CRPR doses had various effects on the blood lipid levels of mice with similar effects to those observed in the MET group. Of note, the CRPR-H group showed best improvement in blood lipid levels. All CRPR doses showed reduced FBG, OGTT, AUC, and HOMA-IR, and the high dose group showed the most obvious reductions, which indicated that CRPR effectively slows down the rapid increase of postprandial blood glucose levels in diabetic mice. These results were consistent with those reported in the literature (28). Thus, the hypoglycemic effect of CRPR may be achieved by lowering the islet cell sensitivity index and improving the IR level in T2DM mice.

A common complication of DM is hyperlipidemia, and diabetic mice can develop dyslipidemia gradually as they age (29). In the present study, CRPR effectively reversed the disordered lipid metabolism of T2DM mice. CRPR may increase HDL-C in serum, which facilitates the transport of cholesterol to the liver and accelerates lipid degradation in the liver, thereby lowering cholesterol levels. Furthermore, liver lipid accumulation aggravates IR and causes a variety of diabetic complications, especially non-alcoholic fatty liver disease (30, 31). A related study has reported that reducing hepatic lipid accumulation restores insulin signaling and reduces IR in mouse livers with T2DM (32). In the present study, CRPR treatment significantly reduced the liver TG and TC levels as well as improved liver lipid accumulation in T2DM mice. Moreover, histopathological analysis of liver sections in the MC group indicated that the hepatocyte structure was disorganized and that some hepatocytes were edematous and large with vacuolar degeneration and local inflammatory cell infiltration, which may contribute to an increased liver index. Unlike hepatomegaly, STZ injection resulted in partial destruction of pancreatic islets and significant atrophy of the pancreas as evidenced by the pancreatic index, and mice in the MC group showed significant islet damage, which affected insulin secretion and reduced FINS values. After CRPR treatment, the number of islet cells significantly increased, and the arrangement was regular with a clear boundary between the glandular vesicles and islets, which indicated a significant improvement, suggesting that CRPR had a certain improvement effect on pancreatic injury, especially in the high-dose group. The present findings were in agreement with those reported by Wang et al. (33).

**Figure 10:**
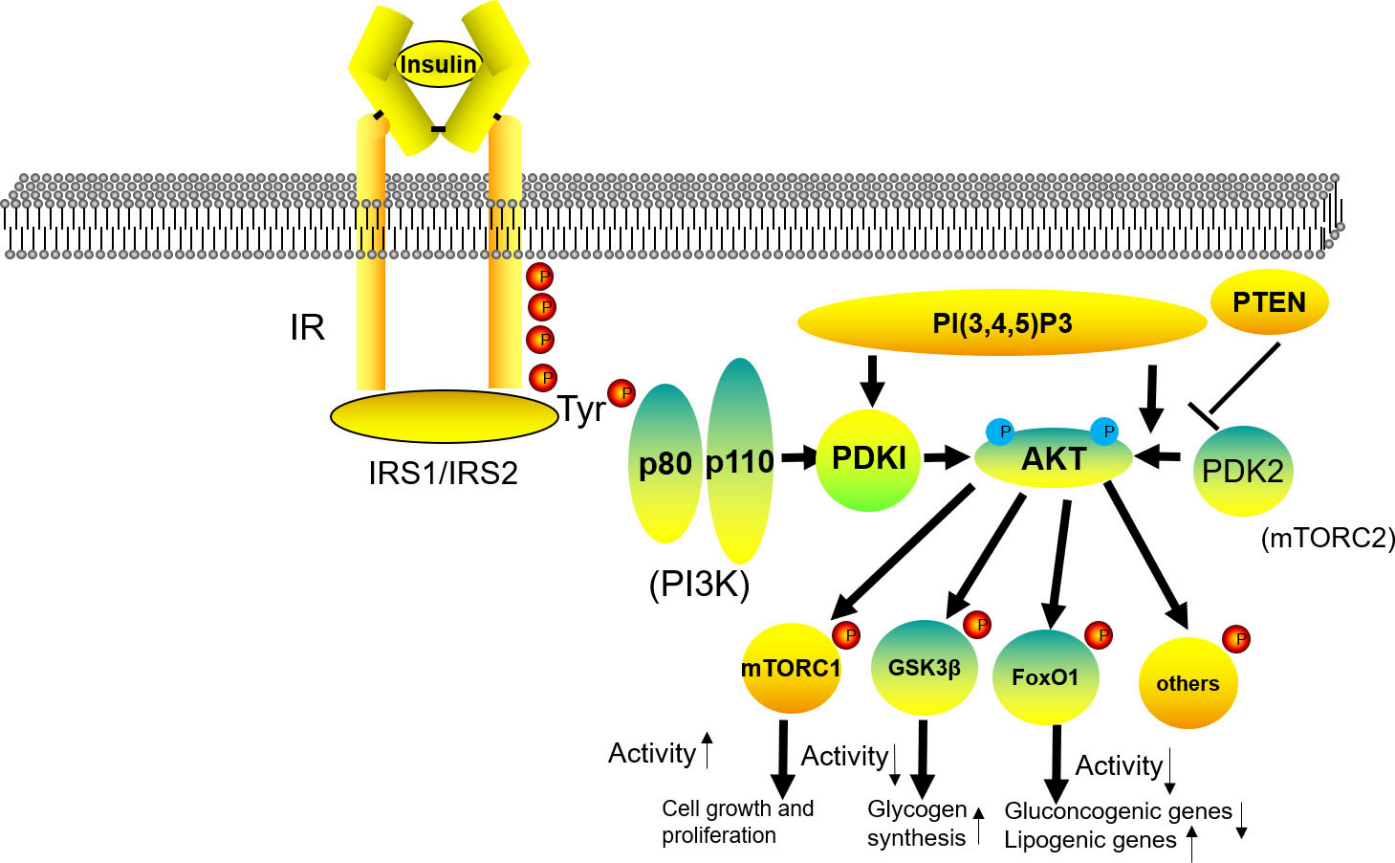
Pharmacological mechanisms of CRPR in the IRS1/PI3K/AKT signaling pathway.

The insulin signaling pathway is a normal physiological functional pathway for insulin to maintain glucose absorption and utilization, and it plays a key role in improving IR. In addition, the PI3K/Akt signaling pathway is a key pathway for insulin to regulate glucose homeostasis and is one of the key pathways for regulating insulin levels (34). Pharmacological mechanisms of CRPR in the IRS1/PI3K/AKT signaling pathway is shown in **Figure 10**. Insulin receptor substrate 1 (IRS1) acts as a signaling protein that connects the inside and outside of cells in the insulin signaling pathway, and its activation strongly influences the physiological effects of insulin (35). Under normal conditions, when insulin binds to surface receptors, IRS1 tyrosine site phosphorylation is activated, which inhibits its serine, and activates the downstream signaling pathway to play a physiological role. However, when IR occurs, IRS1 serine phosphorylation is enhanced, and the downstream signaling pathway is not activated (36). In the present study, compared to the NC group, the expression of p-IRS1 was significantly higher in the MC group, which inhibited insulin signaling and aggravated IR. After CRPR treatment, especially at the high dose, the expression of p-IRS1 protein in liver tissue was decreased, which activated the downstream PI3K/Akt signaling pathway, resulting in increased expression levels of p-GSK3β, GSK3β, PI3K, and p-Akt. It shows that CRPR can effectively restore the phosphorylation level and expression level of the protein, making it tend to be normal. Akt is a downstream molecule of IRS1, and it is also an intersection point in the PI3K/Akt pathway that regulates several signaling pathways that play a key role in glucose metabolism, including regulation of the GSK3β protein (37). GSK3β regulates the activity of glycogen synthase, and when PI3K/Akt is activated, Akt is phosphorylated, which inhibits the expression of GSK3β protein and stimulates glycogen synthesis, ultimately exerting a hypoglycemic effect. Thus, these findings further indicated that CRPR effectively prevents and treats T2DM by activating the IRS1/PI3K/Akt signaling pathway.

In conclusion, CRPR inhibits IR and improves abnormal glucose tolerance and blood lipid levels in a T2DM mouse model to prevent the occurrence of diabetic complications. Moreover, CRPR significantly improves the structure and function of the liver and pancreas in mice, which increases insulin secretion and lowers blood glucose levels. The hypoglycemic mechanism of CRPR may occur *via* the activation of the IRS1/PI3K/Akt signaling pathway to improve IR in T2DM mice as well as by promoting glycogen synthesis and regulating blood glucose levels. However, the mechanism of the downstream pathway needs to be further investigated. Overall, the results of the present study provide data support and methodological reference for the development and mechanisms of related CRPR preparations.

## Data availability statement

The original contributions presented in the study are included in the article/Supplementary Material. Further inquiries can be directed to the corresponding authors.

## Ethics statement

The animal study was reviewed and approved by Animal Experiment Ethics Committee of People’s Hospital Affiliated to Chongqing Three Gorges Medical College. Written informed consent was obtained from the owners for the participation of their animals in this study.

## Author contributions

B-BF and J-HZ proposed the idea and designed the study. Y-PM performed the study and wrote the manuscript. Y-MS, W-XW, and NL interpreted the data and revised the manuscript. All authors contributed to the article and approved the submitted version.
